# Hyperforin Ameliorates Imiquimod-Induced Psoriasis-Like Murine Skin Inflammation by Modulating IL-17A–Producing γδ T Cells

**DOI:** 10.3389/fimmu.2021.635076

**Published:** 2021-05-05

**Authors:** Song Zhang, Jia Zhang, Juanjuan Yu, Xiaolu Chen, Fangyuan Zhang, Wei Wei, Lingyun Zhang, Wenmao Chen, Nengxing Lin, Yan Wu

**Affiliations:** ^1^ Department of Dermatology, Union Hospital, Tongji Medical College, Huazhong University of Science and Technology, Wuhan, China; ^2^ Department of Dermatology, The First People’s Hospital of Jiangxia District, Wuhan, China; ^3^ Department of Neurology, Union Hospital, Tongji Medical College, Huazhong University of Science and Technology, Wuhan, China

**Keywords:** IL-17A, psoriasis, hyperforin, γδT cells, Stat3

## Abstract

Hyperforin is a major active constituent of *Hypericum perforatum* L. extract, which is widely used for the treatment of depressive disorders. Recent studies have reported that hyperforin reduced inflammation in stroke and suppressed proliferation and differentiation in keratinocytes. Psoriasis is a chronic immune-mediated inflammatory skin disease in which the IL-23/IL-17 axis plays an important role. To investigate the underlying inflammatory mechanisms and response of hyperforin in psoriasis, we use imiquimod (IMQ)-induced mice model, *in vitro* cultured murine splenic γδ T cells, and HaCaT cells in this study. Data showed that hyperforin reduced epidermal thickness and decreased IMQ-induced pathological scores of cutaneous skin lesions in mice. Meanwhile we proved that hyperforin suppressed infiltration of CD3^+^ T cells and downregulated expression of *Il1*, *Il6*, *Il23*, *Il17a*, *Il22*, antimicrobial peptides (AMPs) in the skin lesion. Hyperforin significantly inhibited imiquimod-induced splenomegaly, reduced serum levels of TNF-α and IL-6, and IL-17A in splenocytes and draining lymph nodes. Our study also suggested that hyperforin lessened the infiltration of γδ T cell and CCR6^+^ γδ T cells in spleen and lymph nodes. Hyperforin also suppressed the typical psoriasis-like inflammatory responses and the infiltration of IL-17A^+^ cells in dermal γδ T cells of IMQ treated *Tcrd* ^−/−^ mice transferred with γδ T cells. *In vitro* studies, hyperforin reduced the expression and secretion of IL-17A in γδ T cells, and suppressed the activation of MAPK/STAT3 pathways in human keratinocyte HaCaT cells and γδ T cells. In conclusion, hyperforin alleviates IMQ-induced inflammation in psoriasis through suppressing the immune responses exerted by IL-17 A-producing γδ T cells and related cytokines by modulating MAPK/STAT3 pathways. Our study provided a novel therapeutic tragedy for psoriasis by which hyperforin attenuates psoriasis-related inflammatory responses.

## Introduction

Psoriasis is a chronic immune-mediated inflammatory skin disease, which is associated with high prevalence, disfigurement, and comorbid diseases (1). IL-23/IL-17 cytokine axis has been repeatedly confirmed to play the key role in the pathogenesis of human psoriasis (1). Novel biologics, such as Ustekinumab and Secukinumab, have showed good curative effects in moderate-to-severe plaque psoriasis (2). T helper 17 cells (Th17), a subtype of CD4^+^ T cells producing IL-17, were reported to play an important role in psoriasis (3, 4). However, recent studies revealed that other innate immune cells, such as IL-17–producing γδ T cells, also involved in the pathogenesis of psoriasis (5, 6). As a commonly studied transcription factor, STAT3 has recently performed to be crucial in psoriatic-like inflammatory conditions (7). This factor also emerges great influence on the pathogenesis of psoriasis through regulating cytokines including the main IL-23/IL-17 axis (8).

Hyperforin is a major active constituent of *Hypericum perforatum* L. extract, which has antidepressant, bactericidal, anti-inflammatory, antioxidant, and other effects (9–13). Clinical studies have supported the topical use of St. John’s wort as an effective treatment for psoriasis (14, 15), but the specific mechanisms are still not completely explored. Previous study has indicated the effects of hyperforin on keratinocytes. Margarethe Muller had reported that hyperforin modulates the differentiation and proliferation of HaCaT cells and primary cultures of human keratinocytes *via* TRPC6 channels by inducing Ca^2+^ influx (16), which may be partially involved in the pathogenesis of psoriasis. Studies had reported the anti-inflammatory effects of hyperforin in pancreatic β cells, microglia, vascular endothelial cells, and neuronal cells (17–19). However, the specific mechanism of hyperforin on inflammatory response mediated by active immune cells and cytokines in psoriasis has not been perfectly proved.

In this study, we used the imiquimod (IMQ)-induced murine psoriatic models, *in vitro* cultured γδ T cells and HaCaT cells to find out the effects of hyperforin on (1): the change of inflammatory cytokines expression and inflammatory cells infiltration in the skin lesion (2); systemic inflammation of spleen and lymph nodes (3); the quantity of γδ T cells in the spleen and lymph nodes (4); the activation of MAPK and STAT3 pathway in *in vitro* cultured γδ T cells and HaCaT cells. Our study will support the novel therapeutic potential of hyperforin in alleviating psoriasis.

## Materials And Methods

### Reagents and Antibodies

Hyperforin (dicyclohexylammonium salt, Product Code: 19572, Cayman Chemical Company, Ann Arbor, Michigan) were dissolved in dimethyl sulfoxide (DMSO, D8414, Sigma Chemical Co., St. Louis, MO, USA) to make the stock solutions (c = 1.4 mmol/L). Before addition to the cell culture or injection to the mice, working solutions were freshly prepared through diluted. The final concentration of DMSO was less than 0.1% and the cytokinetic parameters were not affected. Chemicals including MTX (CAS: 133073-73-1), LPS (L2880), TNF-α (CAS: 94948-59-1) were obtained from Sigma Chemical Co., St. Louis, MO, USA. Soluble γδTCR antibody (107502), anti-CD28 (102101), and IFN-γ (517904) were obtained from BioLegend. IL-2 (212–12), IL-1β (211-11B), and IL-23 (200–23) were purchased from PeproTech. CD4+ sorting magnetic beads were obtained from Miltenyi Biotec. PCR primers were purchased from Takara Biotechnology, Dalian, China. Anti-human antibodies including STAT3 (ab119352), p-STAT3 (705) (ab76315), ERK (ab32537), p-ERK (ab79483), JNK (ab213521), p-JNK (ab131499), p38 (ab31828), p-p38 (ab178867) were purchased from Abcam Company. Anti-mouse antibodies including STAT3 (AF6294), p-STAT3 (705) (AF3293), ERK (AF0115), p-ERK (AF1015), JNK (AF6318), p-JNK (AF3318), p38 (AF6456), p-p38 (AF4001) were purchased from Affinity Company.

### Animal Use

BALB/c and C57BL/6J mice used in this experiment were purchased from Beijing HFK Bioscience Co., Ltd. *Tcrd* ^−/−^ mice on a C57BL/6J background were kindly donated by Jing Luo. They were then bred in the animal facility under specific pathogen-free conditions for more than a week before the experiment. All mice were age- and weight-matched when used in experiments. Animal experiments were performed in the USUHS laboratory animal facility. The protocol used in these experiments was approved by the USUHS Institutional Animal Care and Use Committee.

### IMQ-Induced Psoriatic-Like Mouse Model

Remove the dorsal hair of mice (8–10 weeks of age) at a surface area of about 4 to 5 cm^2^ as described in our previous publication (13), 5% imiquimod (IMQ) cream (Mingxin Lidi Laboratory, China) was applied daily at a topical dose of 62.5 mg for 7 days to establish IMQ-induced psoriasis mouse model (20).

### Groups of Mice Model

BALB/c mice were randomly divided into the following groups: control group; IMQ treated groups (topical dose with 62.5 mg of 5% IMQ cream alone); MTX treated positive control group (1.0 mg/kg/week, intraperitoneal injection); Hyperforin group (5 mg/kg/day, intraperitoneal injection), as used by others (10). MTX was dissolved in the saline while Hyperforin was dissolved in DMSO. Each group have five mice, and seven consecutive days of administration would be necessary for all mice. γδ T cells (2 × 10^6^) pretreated or not with hyperforin (suspended in 200 μl sterile PBS) were administered into *Tcrd* ^−/−^ mice *via* tail vein injection. Control *Tcrd* ^−/−^ mice were injected with 200 μl PBS. Five days later, for the mouse model, the reconstituted mice were subjected to induction of psoriasis-like disease. All mice were sacrificed, and samples were collected for analysis on day 8.

### Measurement of Skin Inflammation Severity

The Psoriasis Area and Severity Index (PASI) consists of measurements of skin erythema, scale, and thickness. In our previous study (20), we have used this measurement to assess the severity of skin lesions. The specific criteria of PASI scores have been described in detail in our previous studies (20). Mice were evaluated since the first day that IMQ was administrated for seven consecutive days. The thickness of the mouse skin was measured using a micrometer, and the average value was measured three times a day. To measure cellular accumulation and epidermal thickness, surgical specimens of dorsal skin tissues were paraffin-embedded for H&E staining. Paraffin-embedded tissues sections (4 μm) were stained with hematoxylin (Beyotime, China) for 40 s and with eosin (Beyotime, China) for 30 s. The tissue sections were examined under an OLYMPUS light microscope. The thickness of mouse epidermis was measured using Photoshop software in three separated fields of view.

### Immunohistochemistry

Skin tissues were fixed with 4% paraformaldehyde for 48 h and embedded with paraffin. Then samples were cut into 4-μm-thick slides. For immunohistochemistry, sections were sequentially incubated with the primary antibody anti-CD3 (1:100, Abcam) and the secondary antibody HRP anti-rabbit IgG (Maxim), and then the color was developed with diaminobenzidine. Using Image J software to measure the region where CD3^+^ cell accumulate for quantitative analyses and expressed as the percentage of positive cells.

### Cytokine Detection by Enzyme-Linked Immunosorbent Assay (ELISA)

Collect the dorsal skin of the control or experimental mouse, remove the attached connective tissue, then put a 0.1cm^3^ tissue into 1ml of saline. Carefully cut the tissue, and grind it thoroughly in a homogenizer to obtain the tissue suspension. The prepared 10% homogenate was centrifuged at 4 degrees at 3,000 rpm for 15 min, and the supernatant was taken for ELISA detection. The level of IL-17A in the skin of control or experimental groups and in the supernatant of *in vitro* cultured γδ T cells were measured by commercial ELISA kits following the manufacturer’s instructions (MultiSciences, China).

### Reverse Transcription and Real-time PCR Analysis

Collect the dorsal skin of the control or experimental mouse, remove the attached connective tissue, then put a 0.1-cm^3^ tissue into 1 ml of saline. Carefully cut the tissue, and grind it thoroughly in a homogenizer to obtain the tissue suspension. The prepared 10% homogenate was centrifuged at 4 degrees at 3,000 rpm for 15 min, and the precipitate was taken for RT-PCR detection. RT-PCR was performed according to the manufacturer’s instructions. Briefly, total RNA was isolated using TRIzol (Invitrogen, Australia Pty. Ltd.), and cDNA was synthesized using RevertAid First Strand cDNA Synthesis Kit (K1622, Thermo Scientific). Quantitative real-time PCR was performed using the SYBR Green kit (Takara Biotechnology) on a real-time PCR system (StepOnePlus™ Real-Time PCR System, Thermo Scientific). The reaction was performed with a denaturation step at 95°C for 30 s, annealing at 60°C for 30 s, and extension at 72°C for 30 s for 45 cycles. The relative quantity of the target mRNA was normalized to the level of GAPDH mRNA (the internal control).

Primers used in this experiment showed below: IL-1 mice F: GCAACTGTTCCTG AACTCAACT R: ATCTTTTGGGGTCCGTCCAACT IL-6 mice F: GACAAAGCC AGAGTCCTTCAGAGAGA R: GGTCTTGGTCCTTAGCCACTCCTT IL-10 mice F: CACAAAGCAGCCTTGCAGAA R: AGA GCAGGCAGCATAGCAGTG IL-17A mice F: CCTCAGACTACCTCAACCGTTCC R: AGGCTCC CTCTTCAGGACCAG IL-22 mice F: GGTGACGACCAGAACATCCA R: CAGCAGGTCCAGTT CCCCAAT IL-23p40 mice F: AATGTGCCCCGTATCCAGTG R: GAAGATGTCAGAGTCAAGC A GGTG GAPDH mice F: AACTTTGGCATTGTGGAAGG R: ACACATTGGG GGTAGGAACA CRAMP mice F: AGG AGATCTTGGGAACCATGCAGTT R: GCAGATCTACTGCTCCGGCTG AGGTA S100A7 F: GCCTCGCTTCATGGA CAC R:CGGAACAGCTCTGTGATGTAGT S100A8 F: TGCGATGGTGATAAAA GTGG R: GGCCAGAAGCTCTGCTACTC S100A9 F:CACAGTTG GCAACCTTT ATG R:CAGCTGATTGTCCTGGTTTG LL37 human F: GCAGTCACCAGAGGAT TGTGAC R: CACCGCTTCACCAGCCC.

### Flow Cytometric Assays

The mice were sacrificed by cervical dislocation, spleens, axilliary, inguinal lymph node and skin were separated and placed in pre-cooled PBS. Grind the spleen and lymph node, use a 1 mL syringe core to grind the tissue cell mixture through a 70 μM cell screen, place the prepared spleen cell suspension in 5 ml of pre-cooled PBS, centrifuge at 400*g* for 5 min, and discard the supernatant. Resuspend the spleen cells in 10 ml RBC Lysis Buffer (prepo tech 10× RBC Lysis Buffer diluted 10-fold) and resuspend the lymph node cells in PBS, incubate for 10 min, centrifuge the cells at 500*g* for 5 min, and remove the supernatant. Resuspend the spleen cells and the lymph node cells in 100 μl PBS again, add 0.5 μg (1 μl) gamma delta TCR antibody (Catalog #11-5711-81, Invitrogen) and 1 μg (5 μl) CCR6 antibody (Catalog# 50-7196-80, Invitrogen). For the mice skin, cut off the skin sample and gently eliminate the subcutaneous fat. Chop skin samples into small pieces with 10 ml of Collagenase from clostridium histolyticum Type IV (1 mg/ml, Catalog #9001-12-1, Sigma) and DNase I (100 μg/ml, Catalog #10104159001, Roche). Incubate at 37°C for 90 min. Use a 1-ml syringe core to grind the skin cell mixture through a 70-μM cell screen, place the prepared skin cell suspension in 10 ml of pre-cooled PBS, centrifuge, and resuspend the skin cells in 100 μl PBS. Add 0.5 μg (1 μl) γδ TCR antibody (Catalog #11-5711-81, Invitrogen) and 1 μg (5 μl) CD3 antibody (Catalog #70-AM003E07-100, Multisciences) and incubate for 30 min. For intracellular staining, after incubating FIX&PERM MEDIUM (Catalog #70-GAS005/2, Multisciences), add 0.5 μg (1 μl) IL-17A antibody (Catalog# 70-AM0I1704-100, Multisciences) and 1 μg (5 μl) IFN-γ antibody (Catalog# 70-AM0IF05-100, Multisciences). Cell samples were finally administrated utilizing the flow cytometer (FACSCalibur, BD Biosciences).

### Isolation of γδ T Cells and in Experimental Design

The mice were sacrificed by cervical dislocation, spleens were ground into a single cell suspension. Place the prepared spleen cell suspension in Tris-NH4Cl for 5 min, centrifuge at 2,000*g* for 7 min, washed twice with RPMI-1640 medium, and then resuspended in 4 ml of pre-warmed RPMI-1640 medium containing 10% serum. Incubate the single cell suspension to the treated nylon wool column at 37°C for 50 min, the eluted cells are spleen-derived T lymphocytes. The collected T lymphocytes were resuspended in PBS, centrifuged, and then supernatant was discarded. Add 40 μl of pre-chilled PBS sorting buffer containing 0.5% BSA and 2 mM EDTA and 20 μl of CD4^+^ sorting magnetic beads to 1 × 10^7^ cells. After mixing, incubate at 4°C for 10 min, and separate them with a separator and LS column. The isolated CD4^+^ T cells were cultured in anti-mouse γδTCR (10 μg/ml) coated Corning plates at 37°C and 5% CO_2_ for 6 days, and soluble anti-mouse CD28 antibody (1 μg/ml) and IL-2 (2 ng/ml) were added. The model group was supplemented with IL-1β (5 ng/ml), IL-23 (5 ng/ml) and anti-mouse IFN-γ antibody (5 μg/ml). The hyperforin groups were supplemented with IL-1β (5 ng/ml), IL-23 (5 ng/ml), anti-mouse IFN-γ antibody (5 μg/ml), and hyperforin (0.1, 1, 10 μM).

### HaCaT Cells Culture and Experimental Design

Human keratinocyte HaCaT cells were purchased from the China Center for Type Culture Collection and cultured in 1,640 containing 10% heat-inactivated FBS, 100-U/ml penicillin and 100-μg/ml streptomycin. The cells were kept in a cell incubator at 37°C under 5% CO_2_ and 95% humidified atmosphere. Cells were incubated with TNF-α (10, 20 ng/ml) for 4 h to induce psoriatic inflammation. The cells were then incubated with hyperforin (0.1, 1, 10 μM) for another 2 h.

### Western Blotting

Total cell and nuclear lysates were prepared as protocol using the following antibodies: STAT3, p-STAT3 (705), ERK, p-ERK, JNK, p-JNK, p-38, p-p38 followed by incubation with a horseradish peroxidase-conjugated secondary antibody and visualized using a Bio-Rad ChemiDoc XRS Imaging System with an XRS camera (Bio-Rad, Hercules, CA, U.S.A.).

### Statistical Analyses

Statistical comparisons between two groups were performed using a Student’s t test. GraphPad Software Prism 6.0 was used for statistical analysis. P values < 0.05 were considered significant.

## Results

### Hyperforin Ameliorated IMQ-Induced Psoriatic Skin Lesion in Mice

**Figure 1:**
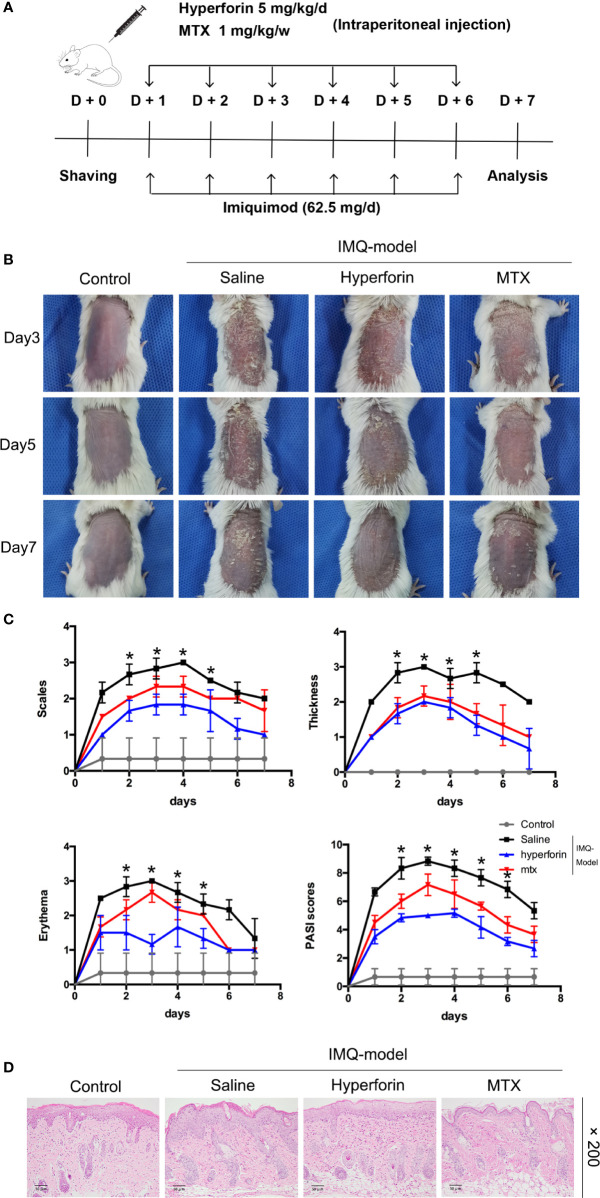
Hyperforin ameliorates psoriatic symptoms and skin inflammation in IMQ-induced psoriatic mice. **(A)** Flow chart of this experiment. **(B)** The back-skin photos of mice were taken at 3th, 5th, 7th day after IMQ painting. **(C)** PASI scores in all groups of mice were evaluated daily and the statistical difference between all groups at 8th day was indicated. **(D)** H&E staining of the dorsal skin with the original magnification of 200×. Data are expressed as mean ± SD (n = 5 mice/group, *p < 0.05 *vs*. Model). One representative of three separate experiments is shown while all results were similar among these three experiments.

To confirm whether hyperforin ameliorated skin lesion in psoriasis, we utilized the IMQ-induced psoriasis-like mice models with or without hyperforin treatment. **Figure 1A** showed the results of this test. IMQ cream was smeared to the shaved back skin of Balb/c mice for eight consecutive days with or without intraperitoneal injection of hyperforin (5 mg/kg/d) and MTX (1 mg/kg/w). As shown in **Figure 1B**, the IMQ treated mice emerged typical psoriasis-like inflammatory responses on back, such as erythema, scaling and thickening, compared to control mice group. Intraperitoneal injection of hyperforin significantly ameliorated skin lesion throughout the treatment period, demonstrated by the reduced severity score of skin inflammation. Therapeutic effect of hyperforin was comparable with MTX, which is effectively used in psoriasis treatment (**Figure 1B**). Administration of hyperforin notably alleviated the severity of IMQ induced psoriasis compared to the IMQ model group according to the scores of erythema, thickness and cumulative score on day 7 (**Figure 1C**). Furthermore, H&E staining suggested that IMQ induced psoriatic lesions, indicated by the presence of epidermal parakeratosis, thickening of acanthosis cell layer, and downward epidermal extension of in-depth dermis. Meanwhile, administration of hyperforin alleviated the severity of the skin lesion (**Figure 1D**). In general, hyperforin exerted similar therapeutic effect in alleviating psoriatic skin lesion compared with MTX (**Figures 1C, D** **)**.

### Hyperforin Inhibited Inflammatory Cell Infiltration and Inflammatory Cytokines Released in Skin of IMQ-Induced Mouse Model

**Figure 2:**
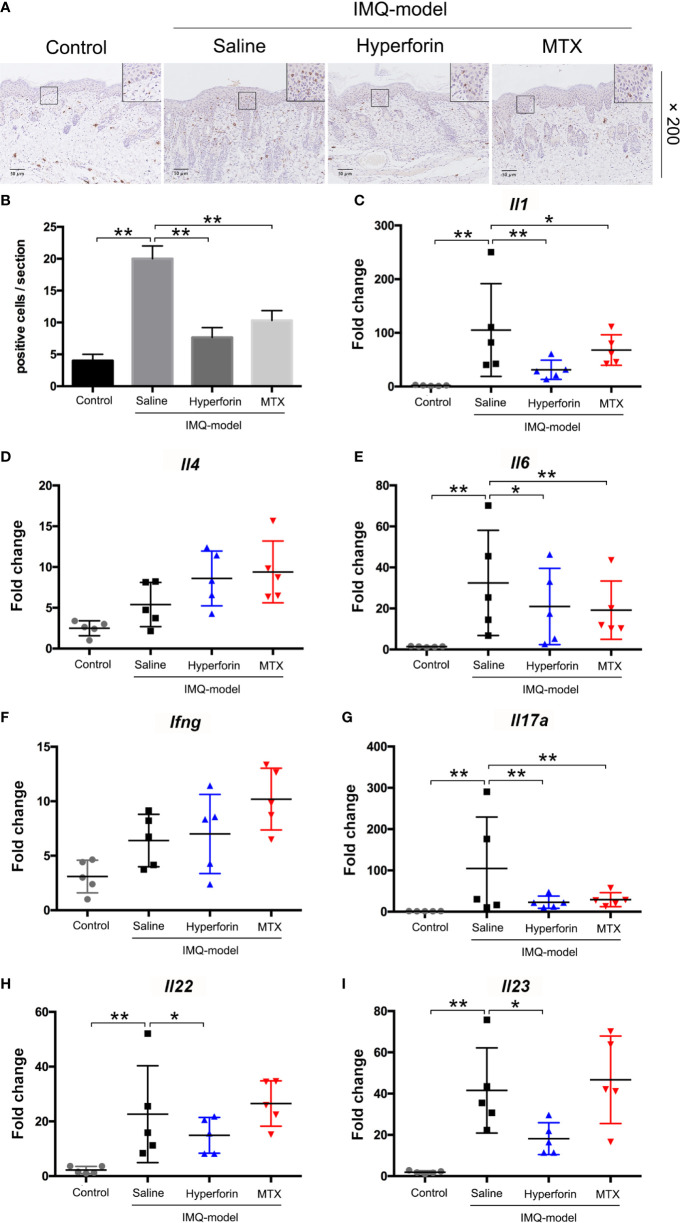
Hyperforin inhibited inflammatory cell infiltration and inflammatory cytokines releasing in skin of IMQ-induced mice model. **(A)** Immunohistochemical staining of CD3^+^ cells in skin lesion. **(B–I)** The mRNA expression of psoriasis associated inflammatory cytokines in the skin lesion of mice model. Data are shown as mean ± SD(A–E). n=5 mice. *P < 0.05 and **P < 0.01 *vs* Model group.

To assess the effect of hyperforin on inflammatory cell accumulation in skin of IMQ-induced mouse model, immunohistochemistry staining of CD3^+^ T cell were performed. Results showed that IMQ induced the accumulation of T cells in dermis and epidermis compared to the control group. Reduced abundance of CD3^+^ T cells in the dermis of hyperforin-treated groups was observed even compared to the MTX group (**Figures 2A, B** **)**.

Furthermore, we tested the mRNA expression of a set of psoriasis related inflammatory cytokines in the skin lesions, and suggested that the mRNA levels of *Il1*, *Il6*, *Il23p40*, *Il17a* and *Il22* in lesions of the model group were highly enhanced, while *Ifng* level showed no remarked change compared with the control group. Also, mRNA level of *Il4* in Th2 cells which exert anti-inflammatory effect did not change markedly. Meanwhile, compared to the model group, the mRNA levels of *Il1, Il6, Il23, Il17a* and *Il22* were decreased in the hyperforin group, of which *Il17a* mRNA declined notably (**Figures 2C–I**).

### Hyperforin Suppressed Imiquimod-Induced Systemic Inflammation

**Figure 3:**
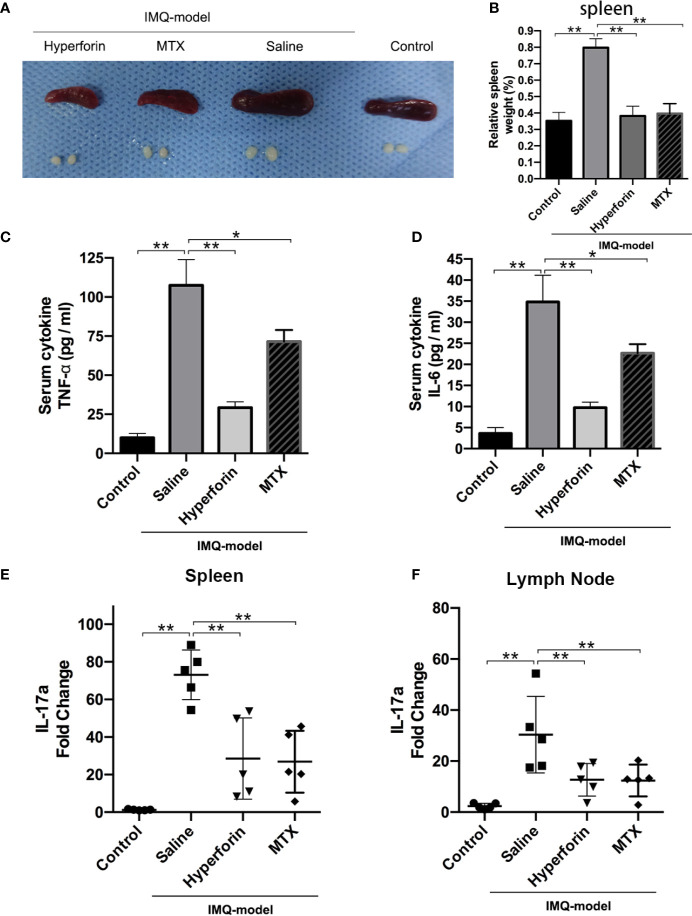
Hyperforin suppressed imiquimod-induced systemic inflammation **(A)** The photos of spleen and skin draining lymph nodes taken at 7th day after IMQ painting. **(B)** Relative spleen weight of the control and test groups. **(C, D)** Serum levels of TNF-α and IL-6. **(E, F)** The mRNA levels of Il17a in spleen and axillary lymph nodes. (n = 5 mice). *P < 0.05 and **P < 0.01 *vs* model group.

As shown in **Figure 3A**, imiquimod increased relative spleen weight, and hyperforin significantly inhibited imiquimod-induced splenomegaly (**Figure 3B**). Hyperforin also significantly inhibited serum levels of TNF-α and IL-6 in IMQ model (**Figures 3C, D** **)**. To evaluate the IL-17A mRNA levels in mice spleen and axillary lymph nodes, **Figure 3E** showed that hyperforin inhibited the IL-17A levels in spleen compared to the model group. Meanwhile, the IL-17A mRNA levels in the axillary lymph nodes exhibited no notable change compared with normal mice (**Figure 3F**).

**Figure 4:**
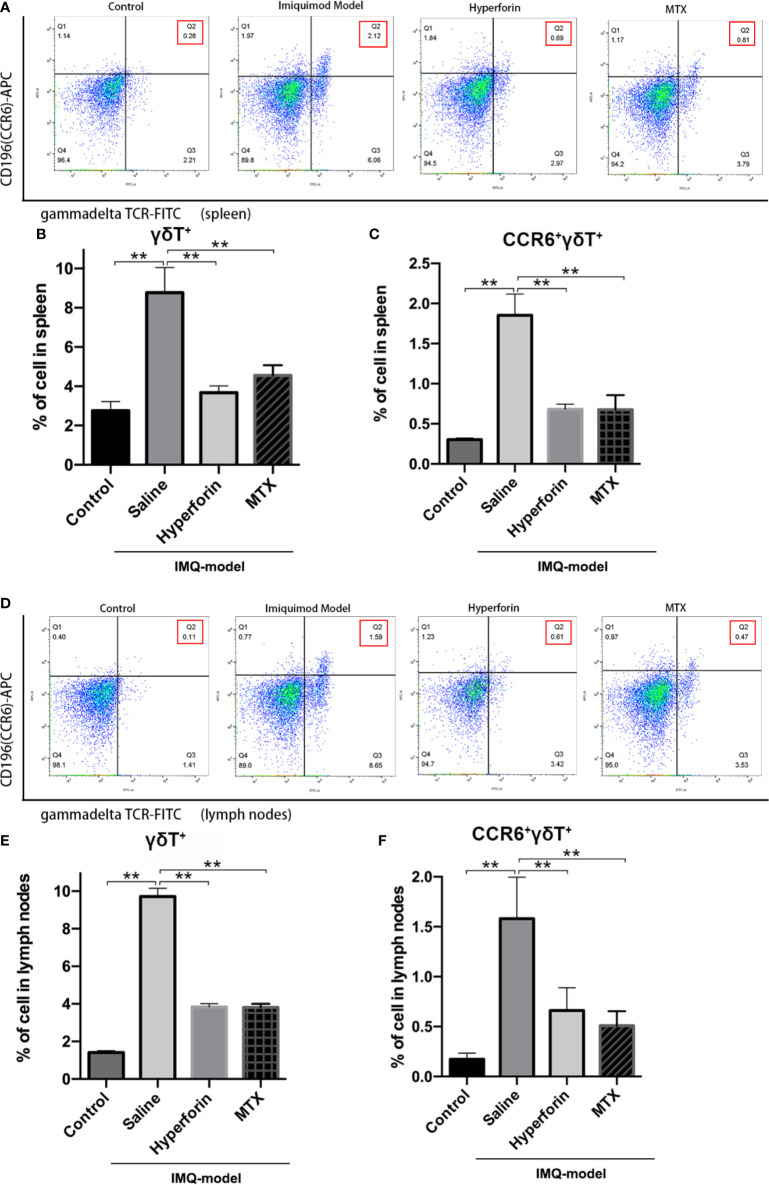
Hyperforin reduced the abundance of γδ T cells in the spleen and lymph nodes of psoriasis-like mouse model. The percentage of γδ T cells and CCR6^+^ γδ T cells in the total live cells in spleen **(A–C)** and axillary lymph nodes **(D–F)**. (n=5 mice). *P < 0.05 and **P < 0.01 *vs* model group.

To further investigate the effect of hyperforin on the infiltration of γδ T cell and CCR6^+^ γδ T cells (the IL-17 A–producing cells), we tested the percentage of γδ T cells and CCR6^+^ γδT cells in spleen and axillary lymph node by utilizing flow cytometry. The ratio of γδ T cells was notably higher in the model group compared to the control group, the ratio of CCR6^+^ γδT cells also accumulated remarkably (**Figures 4A–C**). Cell mass in spleen of the model group shifted toward the right compared to the control group (**Figure 4A**). Lower ratios of γδ T cells and CCR6^+^ γδT cells were presented in the spleen (**Figures 4A–C**) and lymph nodes (**Figures 4D–F**) of hyperforin-treated mice compared to the model group. In general, both hyperforin and MTX inhibited the infiltration of splenic and lymphatic γδ T cells and CCR6^+^ γδT cells.

### Hyperforin Downregulated the mRNA Expression of Antimicrobial Peptides (AMPs) in skin of IMQ-Induced Mouse Model and TNF-α Stimulated HaCaT Cells

**Figure 5:**
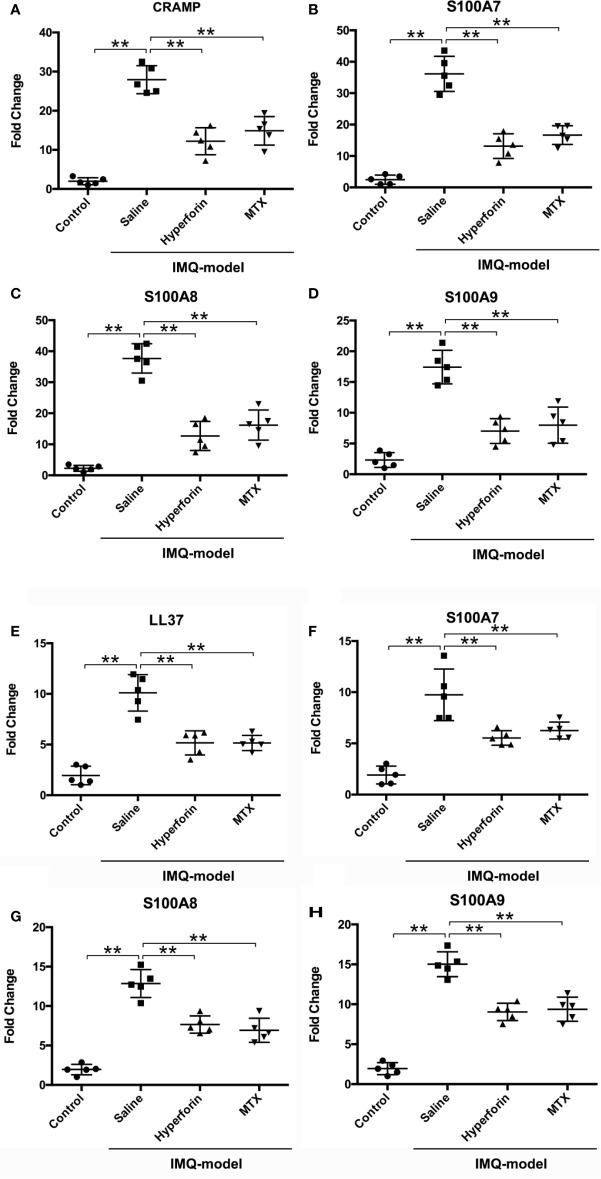
Hyperforin downregulated the mRNA expression of AMPs in in skin of IMQ-induced mouse model and TNF-α stimulated HaCaT cells. The mRNA expression of AMPs in the skin lesion of mice model, CRAMP **(A)**, S100A7 **(B)**, S100A8 **(C)**, and S100A9 **(D)**. After pre-treatment with hyperforin, HaCaT cells were stimulated with TNF-α, and RT-qPCR was used to detect the effects of hyperforin on the mRNA expression of LL37 **(E)**, S100A7 **(F)**, S100A8 **(G)**, and S100A9 **(H)**. Data are expressed as fold induction of relevant mRNA sequences compared to untreated controls. Data represent mean ± SEM from at least three independent experiments performed in triplicates; **P < 0.01 *vs* model group.

Previous study reported that hyperforin has effects on bactericidal and were often used to treat infection (10). Recently, antimicrobial peptides and proteins (AMPs) such as cathelicidin, β-defensins, and S100 proteins, secreted by keratinocytes are inferred to be related with severity of psoriasis lesions (21), and excessive production of AMPs are widely confirmed in psoriasis lesions (21–23). Previous studies have indicated that imiquimod increase the production of AMPs (24–26). Therefore, our experiments were exerted to demonstrate the effect of hyperforin on the expression of AMPs. Data showed a result of remarkably enhanced expression of S100A7, S100A8, S100A9 and CRAMP by imiquimod. In the hyperforin treated group, expression of S100A7, S100A8, S100A9 and CRAMP was highly reduced (**Figures 5A–D**).

To investigate whether hyperforin could directly inhibit the productions of AMPs from *in vitro* cultured keratinocytes, HaCaT cells were stimulated with TNF-α and RT-qPCR was used to detect the effects of hyperforin on the mRNA expression of LL37, S100A7, S100A8, and S100A9. Data showed a result of remarkedly enhanced expression of S100A7, S100A8, S100A9, and LL37 by TNF-α. In the hyperforin treated group, expression of LL-37, S100A7, S100A8, and S100A9 was highly reduced (**Figures 5E–H**).

### Effects of Hyperforin in γδ T Cells on IMQ-Induced Psoriasis-Like Skin Inflammation

**Figure 6:**
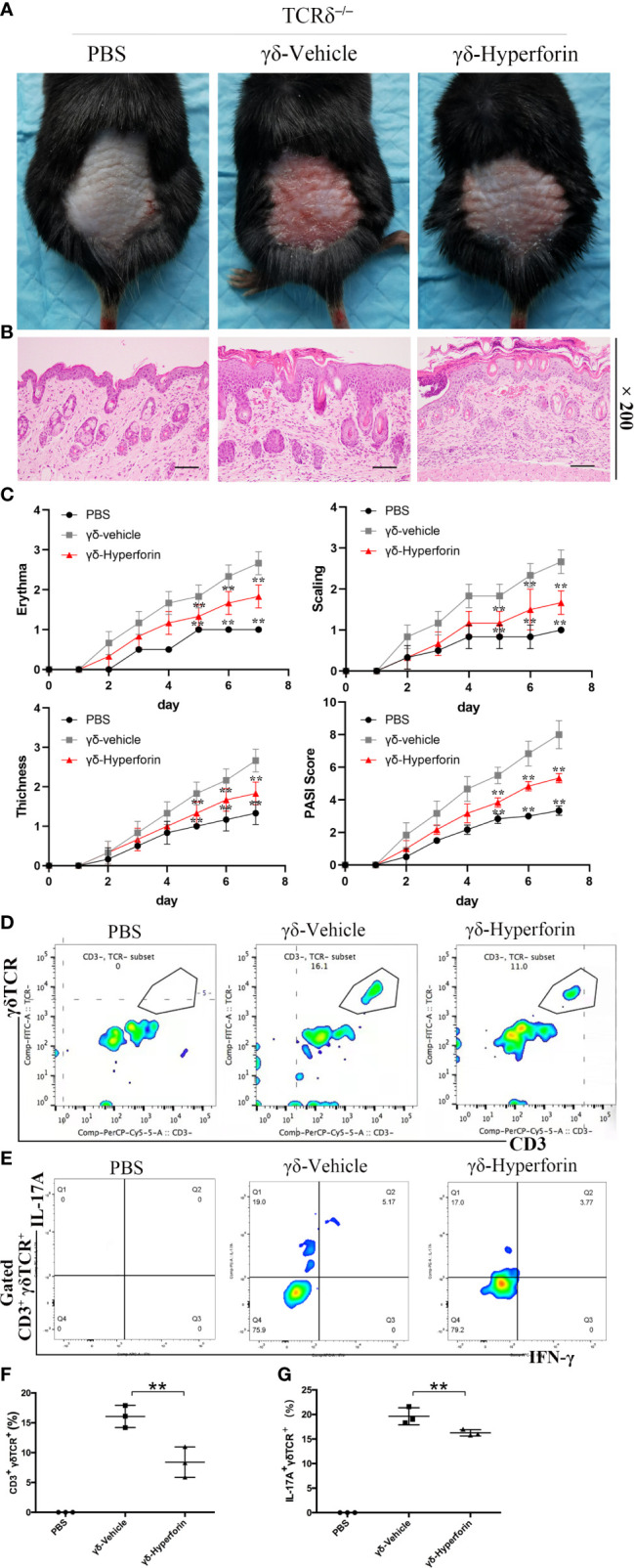
Effects of hyperforin in γδ T cells on IMQ-induced psoriasis-like skin inflammation. *Tcrd* ^–/–^ mice were transferred with PBS or naive γδ T cells pretreated with hyperforin (γδ-Hyperforin) or not (γδ-Vehicle). The mice were then subjected to IMQ-induced psoriasis as part of the psoriasis-like mouse model. The back-skin photos of mice **(A)** and H&E staining of skin lesions **(B)** (scale bars: 100 μm), and PASI scores **(C)**. n = 3. **(D, F)** Detection of the transferred γδ T cells in skin. **(E, G)** Percentage of IL-17A^+^ cells in dermal γδ T cells (gated on CD3^+^ γδTCR^+^ T cells). n = 3. Data are presented as the mean ± SD. (**P < 0.01).

**Figure 6A** showed that the IMQ treated *Tcrd* ^–/–^mice transferred with γδ-vehicle T cells emerged typical psoriasis-like inflammatory responses on back, such as erythema, scaling and thickening, compared to the control group. However, *Tcrd* ^–/–^ mice transferred with γδ-Hyperforin T cells had notably alleviated IMQ-induced skin lesions. H&E staining suggested that IMQ induced typical pathological characteristics of psoriasis in *Tcrd* ^–/–^ mice transferred with γδ-vehicle T cells, such as epidermal parakeratosis, thickening of acanthosis cell layer, and downward epidermal extension of in-depth dermis (**Figure 6B**). However, the pathological characteristics in γδ-Hyperforin group showed reduced epidermal parakeratosis, thinning of the acanthosis cell layer and reduction of the downward epidermal extension of in-depth dermis (**Figure 6B**). *Tcrd* ^–/–^ mice transferred with γδ-Hyperforin notably alleviated the severity of IMQ induced psoriasis compared to the γδ-vehicle T cells group according to the scores of erythema, scaling, thickness and PASI score on day 7 (**Figure 6C**).

In order to detect the transferred γδ T cells in skin, we prepared the single-cell suspensions of the mice trunk skin tissues, and performed the cytofluorimetric analysis of CD3 and γδTCR antigens. The γδ-vehicle group and the γδ-Hyperforin group showed elevated percentages of dermal γδ T cells infiltration (**Figure 6D**). To further observe the infiltration of γδ T17 cells in dermis, we performed the cytofluorimetric analysis of CD3, γδTCR, IL-17A, and IFN-γ antigens. The γδ-vehicle group showed elevated percentage of IL-17A^+^ cells in dermal γδ T cells (gated on CD3^+^ γδTCR^+^ T cells) compared to the γδ-Hyperforin group (**Figure 6E**). The percentage of transferred γδ T cells in skin and IL-17A^+^ cells in dermal γδ T cells (gated on CD3^+^ γδTCR^+^ T cells) were showed in **Figure 6F** and **Figure 6G**. In summary, these findings proved that the abnormal differentiation of γδ T17 cells induced by hyperforin may play an important role in psoriasis.

### Hyperforin Reduced the Expression and Secretion of IL-17A in γδ T Cell *In Vitro*

**Figure 7:**
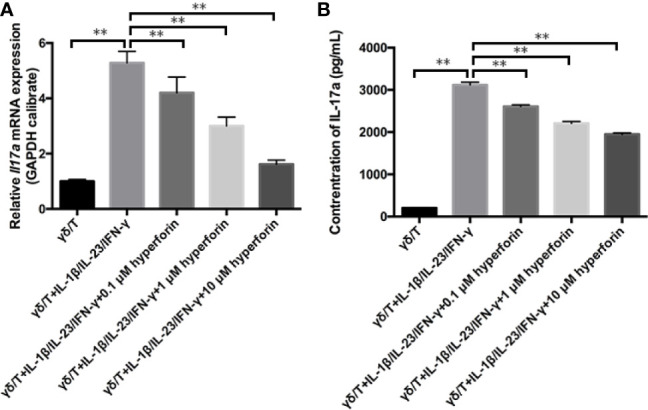
Hyperforin reduced the expression and secretion of IL-17A by γδ T cell *in vitro*. **(A)** The expression of *Il17a* mRNA was significantly lower than that in the model group. **(B)** The level of IL-17 A in the culture supernatant was measured by ELISA to determine the effects of hyperforin on the secretion of IL-17 A by cultured γδ T cells (n=8). **P < 0.01 *vs* model group.

To investigate whether hyperforin has an effect on the function of γδ T cells, we cultured murine splenic γδ T cell *in vitro*. By using the MTT assay, we found that hyperforin, at the concentration of 0.1 to 10 μM, did not affected the viability of cultured γδ T cell (Data not shown). Therefore, we use the concentration range of 0.1 to 10 μM in the subsequent *in vitro* experiments. **Figure 7A** showed that the mRNA level of *Il7a* was increased in the model group compared to the control group, while hyperforin decreased the mRNA level of *Il7a*, and this inhibitory effect was gradually enhanced as the dose increases. Also, we detected the secreted IL-17A in the supernatant by ELISA. **Figure 7B** showed that the supernatant of γδ T cell incubated with hyperforin secreted less IL-17A than the model group.

### Hyperforin Reduced the Phosphorylation of MAPK and STAT3 Pathways in γδ T Cell *In Vitro*

**Figure 8:**
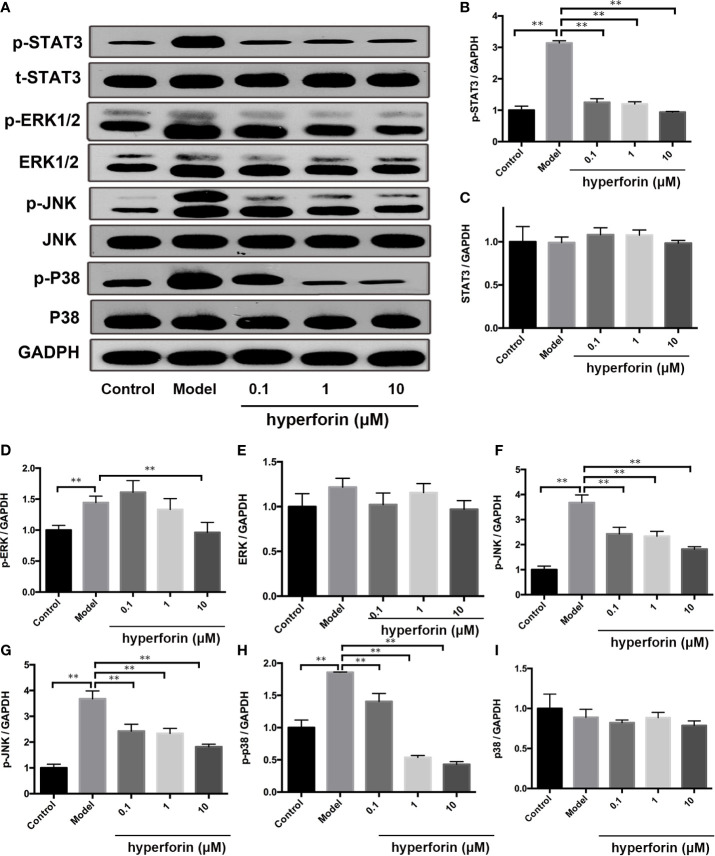
Hyperforin inhibits phosphorylation of MAPK and STAT3 pathway components in *in vitro* cultured γδ T cells. **(A)** Representative images of Western blot. **(B–I)** quantification of the Western blot data by densitometric analysis and normalization to GAPDH (n = 3 independent experiments). **P < 0.01 *vs* model group.

MAPK/STAT3 activation plays an important role in the pathogenesis of psoriasis (27, 28). The expression and phosphorylation of p38, ERK, JNK and STAT3 in the *in vivo* cultured γδ T cells were detected by Western Blot. **Figure 8** showed that the expressions of p-p38, p-ERK, p-JNK and p-STAT3 were increased in the model group compared to the control group, while the expression of p38, ERK, JNK and STAT3 were not changed. Furthermore, hyperforin, especially at the dosage of 10 μM, reduced the expressions of p-p38, p-ERK, p-JNK and p-STAT3.

### Hyperforin Suppressed Phosphorylation of MAPK and STAT3 Pathways in TNF-α Stimulated HaCaT Cells

**Figure 9:**
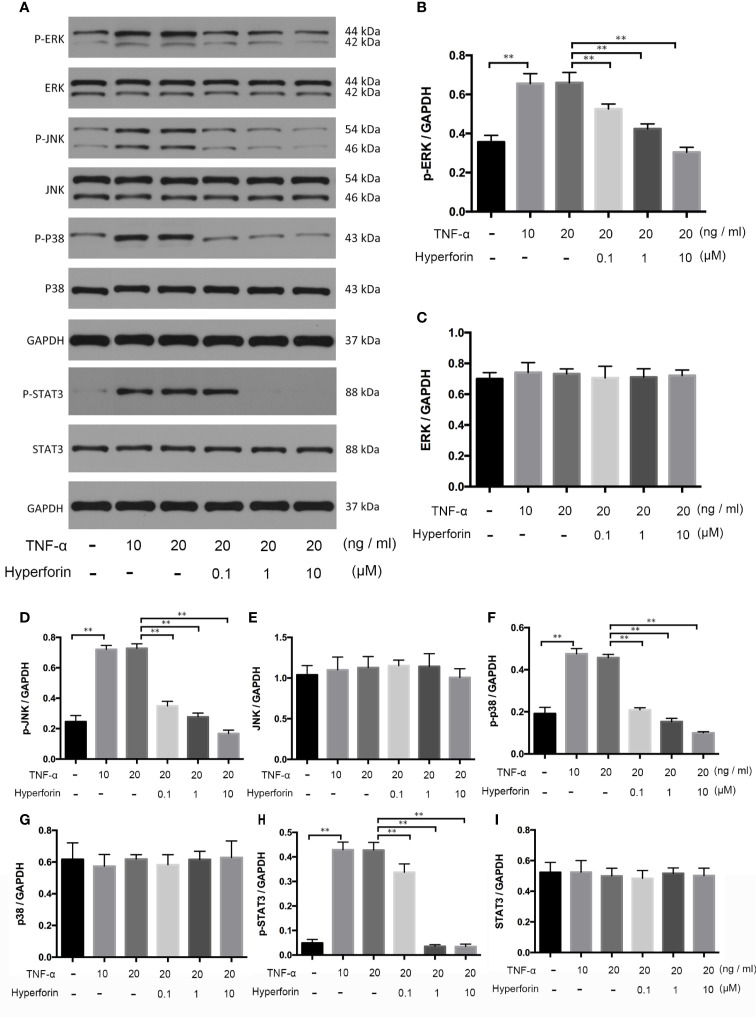
Hyperforin inhibits phosphorylation of MAPK and STAT3 pathway components induced by TNF-α in HaCaT cells. HaCaT cells were pretreated with different doses of hyperforin, and stimulated with TNF-α. The total protein was extracted from the cells and associated protein expression was determined *via* western blotting **(A)**. The quantification data are shown in the right panel **(B–I)**. (n = 3 independent experiments). **P < 0.01 *vs* model group.

To investigate whether hyperforin has an effect on keratinocytes, we cultured TNF-α stimulated HaCaT cells. The expression and phosphorylation of p38, ERK, JNK, and STAT3 in the *in vivo* cultured HaCaT cells were detected by Western blot. **Figure 9** showed that the expressions of p-p38, p-ERK, p-JNK, and p-STAT3 were increased in the TNF-α-stimulated HaCaT cells compared to the control group, while the expression of p38, ERK, JNK, and STAT3 were not changed. Furthermore, hyperforin, especially at the dosage of 10 μM, reduced the expressions of p-p38, p-ERK, p-JNK, and p-STAT3.

## Discussion

In this study, we demonstrated that hyperforin alleviated IMQ induced psoriasiform dermatitis in mice. These mice treated with hyperforin showed lower cumulative scores, epidermal thickening, inflammatory cell infiltration and inflammatory cytokines released in IMQ-induced psoriasis-like mouse model. In addition, hyperforin also reduced enlargement of spleen. Moreover, hyperforin inhibited γδ T cells infiltration in spleen and lymph nodes and showed a similar effect on suppression of epidermal thickening and inhibition of systemic inflammation without obvious side effects compared to MTX which is commonly used for psoriasis (1). *In vitro* study, we found that hyperforin reduced the expression and secretion of IL-17A in γδ T cell. Moreover, we demonstrated that hyperforin significantly inhibited the phosphorylation of MAPK/STAT3 signaling in *in vitro* cultured γδ T cell and TNF-α-stimulated HaCaT cells. In this study, it is the first time we elucidated the reduction effect of hyperforin on γδ T cells. The interplay of immune cells and skin-resident keratinocytes participates in establishing and sustaining inflammatory and immune responses in psoriasis (29). Therefore, our study showed the dual regulation of hyperforin in the keys cells of psoriasis. In conclusion, our results presented a novel mechanism of hyperforin in psoriasis and provided a potential effective approach for psoriasis therapy.

As a specific TRPC6 activator, hyperforin is a phytochemical produced by parts of the members of the plant genus Hypericum (St John’s wort) (9). Previous study reported the expression levels of TRPC6 channel on mRNA and protein levels are significantly reduced both in cultured psoriatic keratinocytes and psoriasis plaques (30). Recently, other group detected mRNA expression levels of TRP channels in PBMCs of 30 patients with psoriasis, data showed that in the patient group, the TRPC6 expression levels were lower compared to controls (31). We have also used the data in the GEO database to perform differential gene analysis to clarify the exact changes of mRNA levels of TRPC6 channels in psoriasis skin lesions. Results elucidated that the mRNA level of TRPC6 in psoriasis skin lesions decreased compared with normal people, however, the statistical difference needs further verification (data not show). Hyperforin is also well-known for its anti-inflammatory, anti-tumor, anti-bacterial, antioxidant, and stabilize skin barrier properties (9–13). Studies had reported the anti-inflammatory effects of hyperforin in pancreatic β cells, microglia, vascular endothelial cells, and neuronal cells (17–19). Previous studies had focused on the modulatory effect of hyperforin in kerationcytes. Topical ointment (5%) in which hyperforin is the main active ingredients, was reported to have anti-inflammatory and anti-psoriatic dermatitis effects (14, 15, 32–34). In our study, it is the first time we applied hyperforin for systemic use to treat psoriasis.

In this study, hyperforin inhibited immune cell activation in psoriasis-like mouse model. To further confirm the valid effect of hyperforin, we examined the mRNA levels of inflammatory cytokine in skin lesions by RT-PCR, the data showed that mRNA levels of *Il1*, *Il6*, *Il23p40*, *Il17a*, and *Il-22* were declined, especially *Il17a*. Recent studies have found that γδ T cells in dermis may be the main source of IL-17A in the skin of imiquimod murine models (5, 6). Moreover, the abundance of γδ T cell and CCR6^+^γδ T were also discovered to change in the spleen of hyperforin treated mice. γδ T cells in spleen performed highly expressed CCR6 and transcriptional factor RORγt (5). Furthermore, we cultured murine splenic γδ T cell *in vivo*. Data showed that hyperforin decreased the mRNA level of *Il7a*, suggesting that hyperforin possibly suppressed γδ T-associated inflammation.

In serum and skin lesions of psoriasis patients, IL-17 and IL-22 were showed to promote the expression of antimicrobial peptides in keratinocytes, such as β-defensin-2 (BD-2), S100A7 (psoriasin), cathelicidin (LL37), and S100A8/9 (calprotectin), all of which may lead to the development of psoriasis in individuals with a higher resistance to skin infections (35, 36). In this study, results showed that hyperforin pretreatment downregulated the mRNA levels of AMPs both in skin lesions of imiquimod-mouse model and *in vitro* cultured HaCaT cells. In psoriasis, decrease of antimicrobial peptides may lead to an increased risk of skin infections (37). In atopic dermatitis, the AMPs expression in the skin lesions are down-regulated, the patients are more susceptible to Staphylococcus aureus skin infections (38). As hyperforin has an anti-bacterial effect, therefore, it may offset the infection risk caused by the decrease in antimicrobial peptides.

Previous studies have suggested that MAPK kinases were involved in the pathogenesis of psoriasis (28). The MAPK kinases constitute three signaling pathways, named mitogen-activated protein kinases p38 (p38 MAPKs), extracellular signal-regulated kinase 1/2 (ERK1/2), and c-Jun N-terminal kinase (JNK) which modulate important functions such as cell proliferation, differentiation, gene expression, and apoptosis within cells (39). Phosphorylation of STAT-3 and of STAT-1 at serine 727 induced by p38 has also been demonstrated in psoriatic lesion (27, 40). Thus, keratinocytes in the psoriatic epidermis are characterized not only by abnormal proliferation and apoptosis but also increased expression of inflammatory cytokines (41). This phenomenon seems to be regulated by the same signal arising from the activation of MAPK signaling cascades of p38 and ERK1/2 (35, 42).

In conclusion, our work demonstrates that hyperforin alleviates IMQ-induced inflammation in psoriasis through suppressing the immune responses exerted by IL-17 A–producing γδ T cells and related cytokines by modulating MAPK/Stat3 pathways. Together with its effectiveness and safety, the current study provides the evidence to support hyperforin as a promising therapeutic tragedy for treatment of psoriasis.

## Data Availability Statement

The original contributions presented in the study are included in the article/supplementary material. Further inquiries can be directed to the corresponding authors.

## Ethics Statement

The animal study was reviewed and approved by the USUHS Institutional Animal Care and Use Committee.

## Author Contributions

SZ, JZ, and JY performed experiment, analyzed data, and wrote the paper. FZ, WW, LZ, and WC performed experiment, analyzed the data, and prepared the images. XC, YW, and NL designed, conducted the research, interpreted data, and wrote the paper. All authors contributed to the article and approved the submitted version. 

## Conflict of Interest

The authors declare that the research was conducted in the absence of any commercial or financial relationships that could be construed as a potential conflict of interest.

## References

[1] Boehncke W-H Schön MP. Psoriasis. Lancet (2015) 386:983–94. 10.1016/S0140-6736(14)61909-7 26025581

[2] Prinz I Sandrock I Mrowietz U. Interleukin-17 Cytokines: Effectors and Targets in Psoriasis-a Breakthrough in Understanding and Treatment. J Exp Med (2020) 217:e20191317. 10.1084/jem.20191397 PMC7037256 31727784

[3] Lowes MA Kikuchi T Judilyn FD Cardinale I Zaba LC Haider AS. Psoriasis Vulgaris Lesions Contain Discrete Populations of Th1 and Th17 T Cells. J Investig Dermatol (2008) 128:1207–11. 10.1038/sj.jid.5701213 18200064

[4] Fitch E Harper E Skorcheva I Kurtz SE Blauvelt A. Pathophysiology of Psoriasis: Recent Advances on IL-23 and Th17 Cytokines. Curr Rheumatol Rep (2007) 9:461–7. 10.1007/s11926-007-0075-1 PMC2893221 18177599

[5] Cai Y Shen X Ding C Qi C Li K Li X. Pivotal Role of Dermal IL-17-producing γδ T Cells in Skin Inflammation. Immunity (2011) 35:596–610. 10.1016/j.immuni.2011.08.001 21982596 PMC3205267

[6] Francisco RV Gray EE Cyster JG. Inflammation Induces Dermal Vγ4+ γδt17 Memory-Like Cells That Travel to Distant Skin and Accelerate Secondary IL-17-driven Responses. Proc Natl Acad Sci USA (2015) 112:8046–51. 10.1073/pnas.1508990112 PMC4491769 26080440

[7] Calautti E Avalle L Poli V. Psoriasis: A Stat3-Centric View. IJMS (2018) 19:171. 10.3390/ijms19010171 PMC5796120 29316631

[8] Schön MP. Adaptive and Innate Immunity in Psoriasis and Other Inflammatory Disorders. Front Immunol (2019) 10:1764. 10.3389/fimmu.2019.01764 31402919 PMC6676248

[9] Shelton RC Keller MB Gelenberg A Dunner DL Hirschfeld R Thase ME. Effectiveness of St John’s Wort in Major Depression: A Randomized Controlled Trial. JAMA (2001) 285:1978–86. 10.1001/jama.285.15.1978 11308434

[10] Fiebich BL Heinrich M Langosch JM Kammerer N Lieb K. Antibacterial Activity of Hyperforin From St John’s Wort. Lancet (1999) 354:777. 10.1016/S0140-6736(05)76019-0 10475222

[11] Meinke MC Schanzer S Haag SF Casetti F Müller ML Wölfle U. In Vivo Photoprotective and Anti-Inflammatory Effect of Hyperforin is Associated With High Antioxidant Activity In Vitro and Ex Vivo. Eur J Pharm Biopharm (2012) 81:346–50. 10.1016/j.ejpb.2012.03.002 22430217

[12] Schempp CM Winghofer B Lüdtke R Simon-Haarhaus B Schöpf E Simon JC. Topical Application of St John’s Wort (*Hypericum Perforatum* L.) and of its Metabolite Hyperforin Inhibits the Allostimulatory Capacity of Epidermal Cells. Br J Dermatol (2000) 142:979–84. 10.1046/j.1365-2133.2000.03482.x 10809859

[13] Chen WT Chen YK Lin SS Hsu FT. Hyperforin Suppresses Tumor Growth and NF-κb-Mediated Anti-apoptotic and Invasive Potential of Non-small Cell Lung Cancer. Anticancer Res (2018) 38:2161–7. 10.21873/anticanres.12457 29599335

[14] Najafizadeh P Hashemian F Mansouri P Farshi S Surmaghi MS Chalangari R. The Evaluation of the Clinical Effect of Topical St Johns Wort (*Hypericum Perforatum* L.) in Plaque Type Psoriasis Vulgaris: A Pilot Study. Australas J Dermatol (2012) 53:131–5. 10.1111/j.1440-0960.2012.00877.x 22571563

[15] Mansouri P Mirafzal S Najafizadeh P Safaei-Naraghi Z Salehi-Surmaghi MH Hashemian F. The Impact of Topical Saint John’s Wort (Hypericum Perforatum) Treatment on Tissue Tumor Necrosis Factor-Alpha Levels in Plaque-Type Psoriasis: A Pilot Study. J Postgrad Med (2017) 63:215–20. 10.4103/0022-3859.201423 PMC5664864 28272075

[16] Müller M Essin K Hill K Beschmann H Rubant S Schempp CM. Specific TRPC6 Channel Activation, a Novel Approach to Stimulate Keratinocyte Differentiation. J Biol Chem (2008) 283:33942–54. 10.1074/jbc.M801844200 PMC2662218 18818211

[17] Novelli M Menegazzi M Beffy P Porozov S Gregorelli A Giacopelli D. St. John’s Wort Extract and Hyperforin Inhibit Multiple Phosphorylation Steps of Cytokine Signaling and Prevent Inflammatory and Apoptotic Gene Induction in Pancreatic β Cells. Int J Biochem Cell Biol (2016) 81:92–104. 10.1016/j.biocel.2016.10.017 27780755

[18] Zhang J Yao C Chen J Zhang Y Yuan S Lin Y. Hyperforin Promotes Post-Stroke Functional Recovery Through Interleukin (IL)-17A-mediated Angiogenesis. Brain Res (2016) 1646:504–13. 10.1016/j.brainres.2016.06.025 27328426

[19] Wang H Shao B Yu H Xu F Wang P Yu K. Neuroprotective Role of Hyperforin on Aluminum Maltolate-Induced Oxidative Damage and Apoptosis in PC12 Cells and SH-SY5Y Cells. Chem Biol Interact (2019) 299:15–26. 10.1016/j.cbi.2018.11.016 30481499

[20] Gan L Duan J Zhang S Liu X Poorun D Liu X. Cold Atmospheric Plasma Ameliorates Imiquimod-Induced Psoriasiform Dermatitis in Mice by Mediating Antiproliferative Effects. Free Radical Res (2019) 53:269–80. 10.1080/10715762.2018.1564920 30663913

[21] Morizane S Gallo RL. Antimicrobial Peptides in the Pathogenesis of Psoriasis. J Dermatol (2012) 39:225–30. 10.1111/j.1346-8138.2011.01483.x PMC3527011 22352846

[22] Lai Y Gallo RL. Amped Up Immunity: How Antimicrobial Peptides Have Multiple Roles in Immune Defense. Trends Immunol (2009) 30:131–41. 10.1016/j.it.2008.12.003 PMC2765035 19217824

[23] Takahashi T Gallo RL. The Critical and Multifunctional Roles of Antimicrobial Peptides in Dermatology. Dermatol Clin (2017) 35:39–50. 10.1016/j.det.2016.07.006 27890236

[24] Eckert RL Broome A-M Ruse M Robinson N Ryan D Lee K. S100 Proteins in the Epidermis. J Invest Dermatol (2004) 123:23–33. 10.1111/j.0022-202X.2004.22719.x 15191538

[25] Nomura I Goleva E Howell MD Hamid QA Ong PY Hall CF. Cytokine Milieu of Atopic Dermatitis, as Compared to Psoriasis, Skin Prevents Induction of Innate Immune Response Genes. J Immunol (2003) 171:3262–9. 10.4049/jimmunol.171.6.3262 12960356

[26] Salamah MF Vallance TM Kodji X Ravishankar D Williams HF Brain SD. The Antimicrobial Cathelicidin CRAMP Augments Platelet Activation During Psoriasis in Mice. Biomolecules (2020) 10:1–13. 10.3390/biom10091267 PMC7565973 32887440

[27] Johansen C Vinter H Soegaard-Madsen L Olsen LR Steiniche T Iversen L. Preferential Inhibition of the mRNA Expression of p38 Mitogen-Activated Protein Kinase Regulated Cytokines in Psoriatic Skin by Anti-Tnfα Therapy. Br J Dermatol (2010) 163:1194–204. 10.1111/j.1365-2133.2010.10036.x 20846304

[28] Andrés RM Hald A Johansen C Kragballe K Iversen L. Studies of Jak/STAT3 Expression and Signalling in Psoriasis Identifies STAT3-Ser727 Phosphorylation as a Modulator of Transcriptional Activity. Exp Dermatol (2013) 22:323–8. 10.1111/exd.12128 23614738

[29] Cristina A Stefania M Paolo G Giampiero G. The Interplay Between Keratinocytes and Immune Cells in the Pathogenesis of Psoriasis. Front Immunol (2018) 9:1549. 10.3389/fimmu.2018.01549 30034395 PMC6043636

[30] Kristina L Margarethe K Ute W Heike B Christian H Boehncke W-H. Reduced TRPC Channel Expression in Psoriatic Keratinocytes Is Associated With Impaired Differentiation and Enhanced Proliferation. PloS One (2011) 6(2):e14716. 10.1371/journal.pone.0014716 21364982 PMC3043053

[31] Özcan S S Gürel G Çakır M. Gene Expression Profiles of Transient Receptor Potential (TRP) Channels in the Peripheral Blood Mononuclear Cells of Psoriasis Patients. Hum Exp Toxicol (2021) 2:960327121991911. 10.1177/0960327121991911 33550865

[32] Haag SF Tscherch K Arndt S Kleemann A Gersonde I Lademann J. Enhancement of Skin Radical Scavenging Activity and Stratum Corneum Lipids After the Application of a Hyperforin-Rich Cream. Eur J Pharm Biopharm (2014) 86:227–33. 10.1016/j.ejpb.2013.06.016 23811220

[33] Takada H Yonekawa J Matsumoto M Furuya K Sokabe M. Hyperforin/HP-β-Cyclodextrin Enhances Mechanosensitive Ca (2+) Signaling in HaCaT Keratinocytes and in Atopic Skin Ex Vivo Which Accelerates Wound Healing. BioMed Res Int (2017) 2017:8701801. 10.1155/2017/8701801 28210627 PMC5292202

[34] Papotto PH Ribot JC Silva-Santos B. Il-17(+) γδ T Cells as Kick-Starters of Inflammation. Nat Immunol (2017) 18:604–11. 10.1038/ni.3726 28518154

[35] Yu XJ Li CY Dai HY Cai DX Wang KY Xu YH. Expression and Localization of the Activated Mitogen-Activated Protein Kinase in Lesional Psoriatic Skin. Exp Mol Pathol (2007) 83:413–8. 10.1016/j.yexmp.2007.05.002 17599830

[36] Wang S Uchi H Hayashida S Urabe K Moroi Y Furue M. Differential Expression of Phosphorylated Extracellular Signal-Regulated Kinase 1/2, Phosphorylated p38 Mitogen-Activated Protein Kinase and Nuclear Factor-Kappab p105/p50 in Chronic Inflammatory Skin Diseases. J Dermatol (2009) 36:534–40. 10.1111/j.1346-8138.2009.00696.x 19785707

[37] Haase I Hobbs RM Romero MR Broad S Watt FM. A Role for Mitogen-Activated Protein Kinase Activation by Integrins in the Pathogenesis of Psoriasis. J Clin Invest (2001) 108:527–36. 10.1172/JCI12153 PMC209397 11518726

[38] Takahashi H Ibe M Nakamura S Ishida-Yamamoto A Hashimoto Y Iizuka H. Extracellular Regulated Kinase and C-Jun N-terminal Kinase are Activated in Psoriatic Involved Epidermis. J Dermatol Sci (2002) 30:94–9. 10.1016/s0923-1811(02)00064-6 12413764

[39] Kyriakis JM Avruch J. Mammalian MAPK Signal Transduction Pathways Activated by Stress and Inflammation: A 10-Year Update. Physiol Rev (2012) 92:689–737. 10.1152/physrev.00028.2011 22535895

[40] Hald A Andrés RM Salskov-Iversen ML Kjellerup RB Iversen L Johansen C. STAT1 Expression and Activation is Increased in Lesional Psoriatic Skin. Br J Dermatol (2013) 168:302–10. 10.1111/bjd.12049 23013371

[41] Arthur JSC Darragh J. Signaling Downstream of p38 in Psoriasis. J Invest Dermatol (2006) 126:1689–91. 10.1038/sj.jid.5700280 16845406

[42] Guan Z Buckman SY Pentland AP Templeton DJ Morrison AR. Induction of Cyclooxygenase-2 by the Activated MEKK1 –> SEK1/MKK4 –> p38 Mitogen-Activated Protein Kinase Pathway. J Biol Chem (1998) 273:12901–8. 10.1074/jbc.273.21.12901 9582321

